# Thoroughness and Honesty in Dental Operations

**Published:** 1875-02

**Authors:** H. H. Townsend


### DENTAL SCIENCE.

Vol. VIII. THIRD SERIES—FEBRUARY, 1875. No. 10.

### ARTICLE I.

### *Thoroughness and Honesty in Dental Operations.*

BY DR. H. H. TOWNSEND.

Read before the Illinois State Dental Society.

It is not my purpose in writing upon this subject to detail any special mode of practice as superior to all others, or applicable in all cases, much less to assume the position of teacher, and attempt to instruct such a body of skillful operators as compose this society, but rather to allude in a general way to some of the more important and frequent deviations from the commonly approved rules of practice. That the *importance* of "thoroughness and honesty in dental operations" is very generally admitted by the dental profession, I believe is true, but that its *observances* is as *generally regarded* in the daily practice of very many operators is, to say the least, doubtful. A law with which every one is familiar, yet, is never enforced, simply exists as a "dead letter" on the statute books. So likewise, the fact that every dentist knows and admits that he ought to be *thorough* and *honest*, avails nothing to those who are too indolent or greedy to practice what they believe to be for the best good of their patients. That there are many in the dental profession who need reforming in this respect, the number of defective operations that almost daily come under our observation will testify, as well as the multitude of useful teeth that are yearly sacrificed to make room for *very false* substitutes.

That some of these men at least know what their duty is in this respect is evident from the fact that they flood our streets with hand-bills informing the public that they do their work *thoroughly* and *honestly*, and lest the truth of their statements be doubted, get their local editors to impress it upon the minds of their readers from time to time, usually calling attention to the *prosperity* of Dr. so and so, and with wonderful sagacity discover among other reasson that the Doctor *understands his business*, and his operations although short and painless, are still *very thorough and honest.* As to being *posted* in their business, if they mean the *advertising* part of it, we fully agree with them, and that they are quick in their operations must also be admitted when they prepare and fill from six to a dozen cavities with gold in an hour, and of course, no one can doubt that a man is a *thorough* operator when he succeeds in finding fourteen cavities in a mouth, where a very superficial examination reveals twenty-five, and that a man is *honest* in his operations requires no further proof than his own statement, that in difficult cases he charges so much for gold filling, patients are compelled to choose amalgam instead. We can, however, hardly consider a man a *thorough* operator, be he never so honest, who passes an instrument out beyond the cervical wall of a proximal cavity until it penetrates the gum which he mistakes for the pulp, and repeatedly attempts to destroy it with arsenic, and after failing to do so, attempts to extirpate it with a barbed broach, and after barbarously tearing off a sufficient quantity to correspond to his idea of the size of a tooth-pulp, proceeds to fill the root, which he does by forcing all the gold he can up into and under the gum; and we cannot wonder that the patient was subsequently opposed to root-filling. We might wonder, perhaps, *why* the people of certain towns *will not pay* for gold filling, and *why* their dentists being ashamed of the quantity of amalgam they use, write letters of apology to the dental depots in consequence; did we not *know* that they tell their patients that the difference between gold and amalgam is, that the latter will preserve teeth a lifetime while gold will last twice as long. Their patients of course, gladly avail themselves of the cheap and easy method of preserving their teeth as long as they live. It would seem however, that a "life time" does not necessarily imply as long as the patient may live, as we usually find that six months, and in some cases as many days even is too long a "life time" for their operations to endure. We know there are those however, who do nothing but thoroughly honest work, and they have achieved very enviable reputations by the excellence of their operations.

They are among the organizers and leaders of this and kindred societies, and not among that class of dentists who require high sounding testimonials to establish their reputation, and the constant and vigorous exertions of their local editors to preserve and protect them, and who really do make one filling of two proximal cavities, erecting a suspension bridge across from one tooth to the other, and treat all difficult cavities, ulcerated teeth, and dental irregularities alike with the forceps. While we recognize the shortcomings of others, let us not like the Pharisee, "thank God we are not as other men," but let us rather become our own critics, and see if at all times, and under all circumstances, we are strictly adhering to our principles of "thoroughness and honesty." Suppose we are operating for a patient from an adjoining town who must return by a certain train, and we are trying to employ every moment to the best possible advantage. The tooth, a second lower molar, is badly decayed; a large cavity in the grinding surface nearly exposes the pulp, while a deep cavity on each proximal surface extends some distance below the gum. The application of the rubber dam, and forcing back the gum, occupied considerable time aud was quite painful, and our patient has become so nervous that it is with great difficulty that we make any progress in the preparation of the cavity, which is exceedingly sensitive. The time is half consumed and still the cavity is not prepared and it will require about all the remaining time to properly fill it. We make another desperate effort and at the end of half an hour find that but little has been accomplished. What is to be done? We suggest a temporary filling and a subsequent sitting, but the patient cannot come again, therefore what we do must be done now. If we give it up we will lose the whole half day, as we are quite certain he will not be willing to pay us for the time already expended unless we complete the operation ; as it is improbable that we can make a perfect operation of it in the limited time now remaining, it becomes a questian whether we shall sacrifice a living tooth, the confidence of our patient, our own honor and integrity for the small fee which we expect to receive for the operation. Suppose however, as we are greatly in need of the money, we decide to reduce the fee in proportion to the quality of the work, and fill it as well as we can under the circumstances, hoping it will at least be worth to him all we charge for the operation. We know there ought to be some kind of a non-conductor used at the bottom of the cavity to protect the pulp from thermal changes, but the time is too limited to attempt it. We therefore place the gold in direct contact with the thin layer of sensitive dentine over the pulp, and proceed to fill as fast as we can, being painfully aware that we are not condensing the gold as thoroughly as it ought to be, especially about the margins, but the filling is as good as the preparation of the cavity, and both correspond to the finish, which consists in passing a separating file down each proximal surface, and slightly burring off the top of the filling, which is being done as we hear the whistle. The patient has just time to pay us our fee and get to the train. Let us suppose we received eight dollars for the operation. We might very appropriately enter on our day book, sold this day for eight dollars "one living tooth, confidence of one patient in dental operations, our own honor and integrity."

For the next few weeks we feel an occasional twinge of conscience, and find ourselves constantly inclined to slight our work. The case is finally forgotten and we once more resume our accustomed habit of thorough operating. Suppose some two years later we have a patient in our chair who belongs to one of the most wealthy and influential families in our town. We find upon examination a dozen or more cavities, but the teeth are of good quality, and the decay is not extensive, and we have no doubt but that they may be preserved by proper treatment. But she is opposed to filling teeth, having had hers poorly operated upon some years previous, and now desires to have them extracted and some artificial ones inserted. We, of course, decline to extract them, and after an extended argument in favor of conservative dentistry, she has about decided to make some appointments and try our efforts for their preservation. At this moment the door opens and our patient of two years previous presents himself, stating "that as he happened to be in town he thought he would just step in and tell us that the tooth we filled for him about a year ago, (they always reduce the time at least one-half) has caused him a great amount of pain ; that the filling came out, and the tooth broke off, and he is very sorry he did not have it pulled out in the first place." Also, "that he has other decayed teeth, but he is going to have them extracted and have false ones." What *fee*, let me ask, would we consider a *sufficient remuneration* for this unhappy result ? Surely, nothing short of a fortune that would enable us to retire from dental practice, and live at our ease.

This, and similar experiences should at least teach us that *hasty manipulation* is not productive of the best results, and that a *reduced* fee can never *compensate* for a *faulty*

*operation*, *poor* work being *dear* at any price. Prof. McQuillen once said that we were justified in charging for the extra time required to do our work thoroughly, but never excusable for negligence of duty. If want of time, or the perverseness of our patients, or any other obstacles interpose to prevent us from doing what our best judgment dictates, we had better decline the operation than to attempt it, and by our failure bring reproach upon ourselves and the profession, which is, of course, judged by the acts of the individual members comprising it. Fortunately such cases are exceptional, but a *reality* nevertheless. It has been my misfortune to meet with two of this kind since our last meeting, both of which I gave up in despair, after exhausting every known means at my command, and patiently and perseveringly spending two half days upon each of them, and really accomplishing nothing. If the very best efforts of the most skillful operators sometimes fail, which is an admitted fact, how can we expect permanent results from our own operations which we *know* are hurriedly and imperfectly performed? And how can we consider ourselves *honest* men if we advise a cheap plastic filling, recommending it to be *just as good as gold*, because the case is a difficult one, or the patient poor, and the next hour when operating on an easy cavity, and for a wealthy patient, advise gold as *the only thing fit to fill teeth with*? Or how shall we justify ourselves as *thorough* operators, by filling cavities in the masticating surfaces of molars and bicuspids, and *guessing* there are no proximal decays. It is our *duty* to deal honestly with our patients, and our *business* to *know* the true condition of the teeth upon which we are operating, and if with the aid of the rubber dam, magnifying mouth mirrors, and quick wedging we are still unable to ascertain the real condition of the proximal surfaces, let us resort to a slower and more effective process of wedging, and if it requires a week's time or more to separate them sufficiently to enable us to obtain the desired information, let us take it, but at all events let us know that the teeth are *sound* before we dismiss them as such. Let us also bear in mind that if we would be consistent as *thorough* and *honest* dentists, we should never dismiss a patient as having no further need of our services, until the whole mouth is in a healthy condition. Not only as already intimated, should all the imperfections of the teeth be searched out and properly cared for, as the requirements, of the case may indicate, but all deposits of tartar or other accumulations should be removed with the utmost thoroughness, not only from the labial surfaces of the anterior teeth, but from *all* the surfaces of *all* the teeth *below* as well as *above* the guns, which, if diseased, should, if possible, by proper treatment, be restored to health.

While this paper is not designed to discuss the relative merits of the different materials employed for filling teeth, yet it seems almost necessary in treating of this subject to refer to the use of amalgam as one of the principal causes of careless and slovenly work. There may be cases when its use is justifiable, but it should be the *exception* and not the rule. I know it is argued by many that the poorer classes must have their teeth saved with something, and they cannot afford gold. But the question arises, *does* amalgam preserve teeth? I think a large majority of the best operators in the profession agree that it makes a very unreliable filling at best.

If this is *true*, we are not saving the teeth of our poorer patients by its use, and *they*, of all our patients, can least afford to pay for an inferior kind of work which is almost certain to fail. The Scriptures tell us that "the destruction of the poor is their poverty," which would seem to be especially the case when they pay their money for poor dental operations, which, when once or twice repeated cost more than would have been paid for good work in the first place, and result in the loss of both money and teeth at last.

Another objection to the use of amalgam is that it unfits the operator for more thorough operations. If "evil communications corrupt good manners," which the good book also tells us is true, we certainly should not cultivate a very intimate acquaintance with this "black visaged imp." I venture the proposition that any one who constantly fills teeth with amalgam three days in the week, will meet with very poor success with the use of gold the balance of the time, should he attempt it. If it is *true* that our patients of limited means cannot afford to pay for cheap operations, it is *also true* that we cannot afford to turn them off with work of an inferior quality. For experience has proven that they are quite as likely to make known our success or failures as are the rich, and in this world of change, and particularly in this free land of ours, no one can predict with any degree of certainty, what will be the financial or social condition of our patients a year hence. The servant of to-day may be mistress of the house to-morrow, and the man of wealth who to-day sits in our chair may be a hod-carrier when next he requires our services. It ever becomes us therefore to try and do good alike to rich and poor, making each operation as perfect as we know how to make it, and if possible an improvement upon the preceding one, and though we may never have an abundance of wealth, we will enjoy the confidence and respect of the majority of our patients, and have the consciousness within our own breasts of having faithfully performed our duty.

